# Transcriptome Analysis of Two Near-Isogenic Lines with Different NUE under Normal Nitrogen Conditions in Wheat

**DOI:** 10.3390/biology10080787

**Published:** 2021-08-17

**Authors:** Xinbo Zhang, Fujian Li, Yonggang Ding, Quan Ma, Yuan Yi, Min Zhu, Jinfeng Ding, Chunyan Li, Wenshan Guo, Xinkai Zhu

**Affiliations:** 1 Jiangsu Key Laboratory of Crop Genetics and Physiology/Jiangsu Key Laboratory of Crop Cultivation and Physiology, Agricultural College of Yangzhou University, Yangzhou 225009, China; zhxb202@126.com (X.Z.); fjli_agriculture@163.com (F.L.); 15949083474@163.com (Y.D.); mq_agriculture@163.com (Q.M.); minzhu@yzu.edu.cn (M.Z.); jfdin@yzu.edu.cn (J.D.); licy@yzu.edu.cn (C.L.); guows@yzu.edu.cn (W.G.); 2 Jiangsu Xuhuai Regional Institute of Agricultural Science, Xuzhou 221131, China; yvonneyi19890803@163.com; 3 Co-Innovation Center for Modern Production Technology of Grain Crops, Yangzhou University, Yangzhou 225009, China; 4 Joint International Research Laboratory of Agriculture and Agri-Product Safety, The Ministry of Education of China, Yangzhou University, Yangzhou 225009, China

**Keywords:** wheat, nitrogen use efficiency (NUE), transcriptome analysis, carbon metabolism, nitrogen metabolism, differentially expressed genes (DEGs)

## Abstract

**Simple Summary:**

High nitrogen use efficiency (NUE) in wheat (*Triticum aestivum* L.) is the key to ensure high yield and reduce pollution. Understanding the physiological and molecular changes that regulate NUE is important for the breeding of high-NUE wheat varieties. Carbon and nitrogen metabolism are the basic metabolic pathways in plants. It becomes important to reveal the underlying molecular mechanisms related to carbon and nitrogen metabolism, which may be helpful to improve NUE. In this paper, two wheat near-isogenic lines (NILs) with contrasting NUE were performed RNA-Sequencing (RNA-Seq) to identify candidate genes associated with carbon/nitrogen metabolism under normal nitrogen conditions. Our research may provide new insights into the comprehensive understanding of the molecular mechanism underlying NUE.

**Abstract:**

Nitrogen (N) is an essential nutrient element for crop productivity. Unfortunately, the nitrogen use efficiency (NUE) of crop plants gradually decreases with the increase of the N application rate. Nevertheless, little has been known about the molecular mechanisms of differences in NUE among genotypes of wheat. In this study, we used RNA-Sequencing (RNA-Seq) to compare the transcriptome profiling of flag leaves at the stage of anthesis in wheat NILs (1Y, high-NUE, and 1W, low-NUE) under normal nitrogen conditions (300 kg N ha^−1^, corresponding to 1.6 g N pot^−1^). We identified 7023 DEGs (4738 upregulated and 2285 downregulated) in the comparison between lines 1Y and 1W. The responses of 1Y and 1W to normal N differed in the transcriptional regulatory mechanisms. Several genes belonging to the *GS* and *GOGAT* gene families were upregulated in 1Y compared with 1W, and the enhanced carbon metabolism might lead 1Y to produce more C skeletons, metabolic energy, and reductants for nitrogen metabolism. A subset of transcription factors (TFs) family members, such as ERF, WRKY, NAC, and MYB, were also identified. Collectively, these identified candidate genes provided new information for a further understanding of the genotypic difference in NUE.

## 1. Introduction

Nitrogen (N) is a key macronutrient for plant growth and development [1]. In recent decades, the extensive use of nitrogen fertilizer has played a key role in increasing crop yield [2]. However, plants can only use 30–40% of the total applied nitrogen fertilizer, more than 60% of the N fertilizer is lost to the atmosphere, groundwater, and rivers, which have seriously influenced the environment [3]. Besides, the heavy use of nitrogen fertilizer increases the cost of production and even reduces the yield [4]. Thus, decreasing the nitrogen application and increasing nitrogen use efficiency (NUE) is necessary for agriculture sustainability.

NUE is often defined as the grain yield produced per unit of applied fertilizer N, and the molecular mechanisms of the NUE traits are complex [5]. NUE is a genetically controlled trait that varies greatly among different crops, such as wheat [6] and rice [7]. It has been reported that NUE is the result of the coordination of carbon and nitrogen metabolism [8]. The carbon metabolism mainly includes the glycolysis, tricarboxylic acid cycle (TCA), and pentose phosphate pathway (PPP); nitrogen metabolism mainly includes nitrogen uptake, nitrate reduction, and amino acid metabolism [9]. Carbon metabolism and nitrogen metabolism in plants are closely related [10]. Carbon metabolism can provide carbon skeletons, energy, and reductants for nitrogen metabolism, while nitrogen metabolism can promote photosynthetic pigments and enzymes for carbon metabolism [11,12]. When the nitrate is assimilated in leaves, it is reduced to ammonium quickly by nitrate reductase and nitrite reductase. The ammonium is then converted into glutamine and glutamate via the glutamine synthetase–glutamate synthase (GS-GOGAT) cycle [13]. The products are converted into other nitrogen-containing compounds for carbon metabolism [14], whereas this cycle requires the carbon skeleton for assimilating nitrogen into amino acids, which derives from the intermediate of carbon metabolism [15]. Therefore, reasonable regulation of carbon and nitrogen metabolism could be an effective way to improve NUE.

Genome-wide expression analysis is an effective method to understand complex traits such as NUE [16]. RNA sequencing (RNA-Seq) is an efficient functional genomics tool and has replaced microarray, which can measure transcript levels more systematically [17]. In recent years, RNA-Seq analysis has been extensively used to explore the molecular mechanisms of NUE at the transcriptome level, researchers have identified genes and regulatory networks in plants [18]. For instance, the transcription factor CDF3 is induced by N starvation in Arabidopsis, which is an important modulating factor for the nitrate response and has the potential to improve NUE in crops [19]. Sinha et al. [20] studied root and shoots transcriptome response to chronic N starvation using two rice genotypes differing in LN tolerance and identified the N-responsive genes that belonged to starch and chloroplast metabolism, which might serve as a resource in enhancing NUE. In another study, Sultana et al. [21] showed that the genes encoding beta-1,3endoglucanase were downregulated in high-NUE wheat cultivar leaves under a N deficiency. In addition, more than 2000 genes were differentially expressed when the N supply was decreased from 2 to 0.2 mM in wheat seedlings. Meanwhile, low N stress markedly increased the transcript levels of genes involved in the signal transduction, carbon/nitrogen metabolism, and antioxidant activity [22]. Likewise, transcriptome analysis on different tissues of durum wheat response to N starvation during grain filling also revealed a large number of genes responsible for photosynthesis, followed by carbon and nitrogen metabolism [23]. Most of the studies focused on the transcriptome responses of plants to N deficiency, and normal N conditions are likely to be present as controls. In the field production of wheat, N fertilization is required to keep the plants growing vigorously and thus achieve high yields. Therefore, transcriptome analysis at normal N application levels is particularly important to improve wheat NUE.

The use of near-isogenic lines (NILs) in transcriptome analysis can reduce genetic background noise. In this study, we investigated the differences in carbon and nitrogen metabolism at the transcriptome level in two wheat NILs (1Y and 1W) under normal N conditions. The major objectives were to: (i) explore the morphological and physiological difference; (ii) screen the DEGs associated with the carbon and nitrogen metabolism by RNA-Seq; (iii) understand the molecular mechanisms of high-NUE in wheat cultivars.

## 2. Materials and Methods

### 2.1. Plant Materials and Experimental Design

Two wheat varieties, P7001 and P216, were used as parents to construct the NILs. The wheat genotype P7001 was selected due to its high NUE compared to other wheat varieties but susceptible to diseases, and the P216 was chosen because it is a commonly grown wheat. The NILs were generated by crossing, back-crossing, and selfing. This experiment used a one-way randomized group design. 1W and 1Y were selected as the materials, and the NUE was 33.41% and 49.33%, respectively (Table 1). Both materials were grown under normal N supply (N1: 300 kg ha^−1^ N, corresponding to 1.6 g N pot^−1^). No nitrogen application (N0) was also used as a control. The experiment consisted of four treatments that were replicated 10 times in 40 pots, and these pots were arranged in four rows.

The experiment was carried out at the Agricultural College of Yangzhou University’s Agricultural Experiment Station (32°23′ N, 119°25′ E) in China. A total of 1.6 g pot^−1^ of N was applied in a split of 5:1:4 at the stage of pre-sowing, four-leaf, and jointing. At the same time, each pot also received an application of single superphosphate (150 kg ha^−1^ P_2_O_5_, corresponding to 0.8 g) and potassium chloride (150 kg ha^−1^ K_2_O, corresponding to 0.8 g) fertilizers respectively before sowing. Twelve wheat seeds were sown by hand on 31 Oct 2019 in plastics pots with a diameter of 26 cm and a height of 26.5 cm. At the three-leaf stage, the seedlings were removed manually to achieve a plant density of 8 plants pot^−1^. Each pot was filled with 12 kg of soil. The soil was loam, and the basic characteristics of the soil were as follows: organic C 16.4 g kg^−1^, available N 117.4 mg kg^−1^, available P 54.8 mg kg^−1^, available K 124.3 mg kg^−1^, and pH 7.26.

### 2.2. Dry Matter and Amount of Nitrogen

Five pots of wheat were harvested at the stage of anthesis and maturity, respectively. The plants were separated into leaf blades, stems and sheaths, and spikes. Then, all samples were heated in an oven at 105 °C for 30 min, dried at 80 °C to a constant weight for biomass and N content measurements. Nitrogen content was determined by the Kjeldahl method [24] using a Kjeldahl apparatus (Kjeltec 8400, FOSS, Hoganas, Sweden). The NUE was calculated as follows:

NUE (%) = (TN_F_ − TN_0_)/N_fertilizer_ × 100

TN_F_ and TN_0_ are the total nitrogen contents of plants in the nitrogen fertilizer and non-nitrogen fertilizer treatment at harvest, respectively, and N_fertilizer_ is the total amount of nitrogen applied.

### 2.3. Net Photosynthetic Rate (Pn)

At the anthesis stage, the net photosynthetic rate (*Pn*) of flag leaves was measured from 09:30 a.m. to 11:30 a.m. on a sunny day using a portable, open-flow photosynthetic system (Li-COR LI-6400, Lincoln, NE, USA). The CO_2_ concentration was set at 380 μmol mol^−1^, and the photosynthetic active radiation (PAR) was set at 1200 µmol m^−2^ s^−1^ [25].

### 2.4. GS and GOGAT Activity Measurement

The flag leaves of the 1Y and 1W were collected at the anthesis stage with three biological replications, and the samples were put into liquid nitrogen rapidly and stored at −80°C before analysis. GS and GOGAT activity of leaves were determined using the GS and GOGAT enzyme kits (Cominbio Biotech, Suzhou, China), respectively. Meanwhile, these flag leaves were used to perform RNA-Seq.

### 2.5. RNA Extraction and Illumina Sequencing

Total RNA was extracted from 0.1 g of flag leaves per sample using Total RNA Kit (Tiangen, Beijing, China), following the manufacturer’s instructions. The purity and integrity of RNA were examined by a nanodrop spectrophotometer (Thermo Scientific, Waltham, MA, USA), agarose gel electrophoresis (Invitrogen, Waltham, MA, USA), and Agilent Bioanalyzer 2100 system (Agilent Technologies, Santa Clara, CA, USA). Total RNA from each sample was used for Illumina sequencing. Sequencing libraries were constructed using Illumina TruSeqTM RNA Sample Preparation Kit (Illumina, San Diego, CA, USA) according to the manufacturer’s specification. Each sample included three independent biological replicates, and then six cDNA libraries were constructed for transcriptome analyses. The libraries were sequenced based on the Illumina HiSeq^TM^ platform, and 150 bp pair-ends reads were generated. To obtain high-quality reads, reads with the adapter, reads with more than 5% unknown nucleotides (N), and low-quality reads (number of bases with Q ≤ 19 more than 50%) were removed.

### 2.6. Transcriptome Sequencing Analysis

The high-quality reads were mapped to the wheat “Chinese Spring” reference genome [26] using HISAT2 (version 2.1.0) software [27]. The public databases were used to predict the functions of the DEGs, including NCBI non-redundant protein sequences database (Nr), NCBI non-redundant nucleotide sequences database (Nt), Universal protein sequence database (UniProt), Protein family database (Pfam), Gene Ontology (GO), and Kyoto Encyclopedia of Genes and Genomes (KEGG). In addition, we also used Plant TF Database v4.0 [28] to identify the transcription factors (TFs). FPKM (Fragments per kilobase of transcript per million mapped reads) method was used to calculate the gene expression levels [29]. Differential expression analyses across samples were conducted in the DESeq2 R package (1.16.1) to identify DEGs [30]. The genes with an absolute value of log_2_FoldChange ≥ 1 and *p*-value < 0.05 were considered as significantly differentially expressed [31]. Gene Ontology (GO) terms with corrected *p*-value < 0.05 were considered to be significantly enriched in DEG analysis [32]. The DEGs were then obtained from three levels: biological process (BP), cellular component (CC), and molecular function (MF) [33]. Besides, pathway analysis was conducted using the Kyoto Encyclopaedia of Genes and Genomes (KEGG) database (https://www.genome.jp/kegg/ (accessed on 29 March 2021)).

### 2.7. Quantitative Real-Time PCR (qRT-PCR) Analysis

Leaf samples were collected at the six-leaf stage and put into liquid nitrogen quickly. 1Y and 1W wheat seedlings samples were used to perform qRT-PCR, with three biological replicates for each N treatment. Total RNA was extracted from leaves of the 1Y and 1W wheat NILs using a Total RNA Kit (Tiangen Biotech, Beijing, China). The first-strand complementary DNA (cDNA) was synthesized by reverse transcription using the HiScript^®^ QRT Super Mix (+gDNA wiper) (Vazyme Biotech, Nanjing, China) according to the manufacturer’s protocol. The first-strand cDNA was used as the template for the PCR. Real-time reverse transcription-polymerase chain reaction (qRT-PCR) amplifications were conducted with ChamQ Universal SYBR^®^ qPCR Master Mix (Vazyme Biotech, Nanjing, China) on the CFX96TM Real-Time PCR Detection System (Bio-Rad, Hercules, CA, USA). Three replicates were conducted for each gene. *GAPDH* was used as an internal reference to check the fold change in the expression of the target genes in all analyses. The primers were designed using Primer Blast (http://www.ncbi.nlm.nih.gov/tools/primer-blast/ (accessed on 5 January 2021)), and the primer sequences were listed in Table S1. The relative expression value of the target genes was calculated using the 2^−^^△△Ct^ method [34]. Verification of the selected DEGs was performed as described above to confirm the reliability of the sequencing data.

### 2.8. Statistical Analysis

All dates were analyzed using the statistical software SPSS 19.0 (SPSS, Inc, Chicago, IL, USA). The least significant difference (LSD) test at the 0.05 level was used to compare 1Y and 1W. Sigma Plot 10.0 software (Systat Software, Inc., San Jose, CA, USA) was used to plot the graphs.

## 3. Results

### 3.1. Effect of Normal Nitrogen on Agronomic Traits of Two Wheat NILs

To determine characteristics of high- and low- NUE wheat NILs under standard N conditions (1.6 g pot^−1^), we sampled five pots plants at the stage of maturity. As shown in Table 1, 1Y had a higher NUE than 1W, and the grain yield in 1Y was 1.22 times significantly higher than 1W. The total N accumulation in 1Y was 1.46 times significantly higher than 1W.

**Figure 1:**
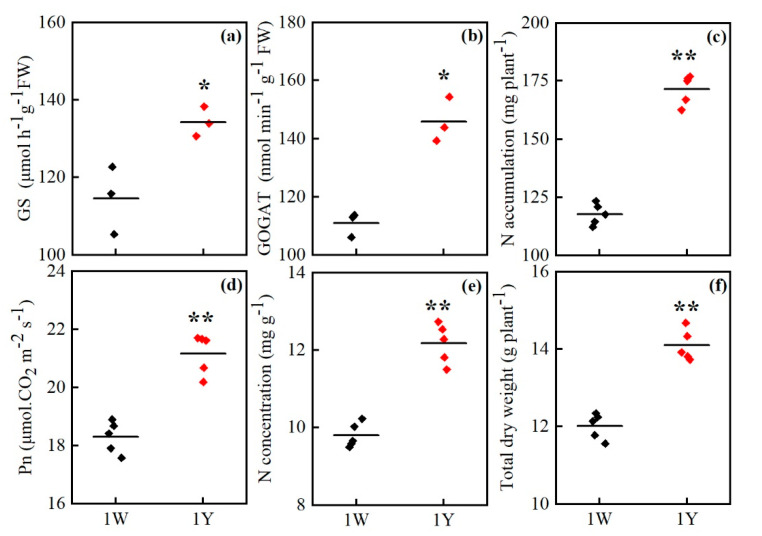
Growth performances of the two wheat genotypes at the anthesis stage under normal nitrogen conditions. Results are shown as means ± SD. (**a**) GS activity; (**b**) GOGAT activity; (**c**) N accumulation; (**d**) net photosynthetic rate (*Pn*); (**e**) N concentration; (**f**) Total dry weight. ♦ and ♦ refer to the replicates of 1W and 1Y, respectively. * and ** mean the significant difference at the *p* < 0.05 and *p* < 0.01, respectively.

To further determine characteristics of high- and low-NUE wheat NILs, we measured growth performance at anthesis. As shown in Figure 1, compared with 1W, 1Y showed higher activity of GS and GOGAT in leaves at the anthesis stage. The activity of GS and GOGAT in 1Y was 1.17 and 1.31 times higher, respectively, than 1W. 1Y had a significantly higher leaf N concentration than 1W under normal nitrogen conditions, and the total N accumulation of 1Y was 1.58 times higher than that of 1W. The net photosynthetic rate (*Pn*) in 1Y was 1.11 times significantly higher than 1W. Besides, the total dry weight in 1Y was increased by 15% compared with 1W. These results suggested that 1Y had strong nitrogen accumulation and material production capacity.

### 3.2. Transcriptome Sequencing Results

In the present study, we compared transcriptome-wide gene expression between 1Y and 1W under normal N conditions at the stage of anthesis. 1Y samples and 1W samples engendered an average of 45.01 million raw reads and 41.40 million raw reads, respectively (Table S2). Among them, the Q30 (%) was more than 92.85%, indicating that the sequencing results were valid. After filtering some low-quality reads, approximately 38.74 to 55.50 million clean reads were acquired from the two groups of samples. The percentage of clean reads was more than 99.31%. In the 1Y group, approximately 36.84 to 52.97 million clean reads were mapped to the reference genome (Total Mapped), and the mapped reads ranged from 95.10% to 95.44%, with an average of 95.32%. The percentage of the sequence aligned to only one position (Uniquely Mapped) was 94.94%, 94.08%, and 94.09. The respective mapped reads information in the 1W group were: 94.76% to 95.33% total mapped; 94.52% to 95.03% uniquely mapped, respectively (Table S2). We determined that the transcriptome data was appropriate for subsequent analysis.

### 3.3. Analysis of Differentially Expressed Genes (DEGs)

**Figure 2:**
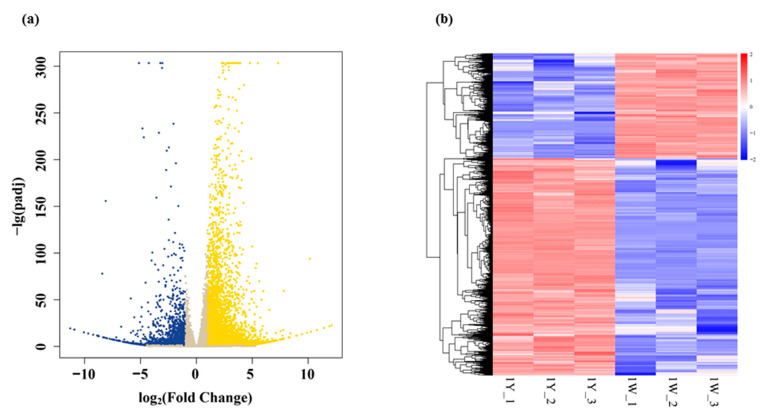
Classification analysis of differentially expressed genes. (**a**) Volcano plots of DEGs between 1Y and 1W. The yellow color indicates the upregulated DEGs, the blue were downregulated genes, and the grey color in the middle indicates the non-significantly genes. (**b**) Clustering analysis of DEGs in the wheat NILs. The x-axis represents different samples. 1Y_1, 1Y_2, 1Y_3 refer to the three replicates of high-NUE 1Y; 1W_1, 1W_2, 1W_3 refer to the three replicates of low-NUE 1W. The y-axis represents the differential genes expressed. Different colors show the genes and the expression levels of the genes are displayed by a color gradient from low (blue) to high (red).

A total of 70,889 genes were identified in the 1Y vs. 1W comparison, of which 7023 genes were differentially expressed, accounting for 9.9% of all these genes. The FPKM values of all genes were compared, and there were significant correlations between replicates (Figure S1). In the current study, 4738 DEGs (67.46% of 7023) were upregulated, and 2285 DEGs (32.54% of 7023) were downregulated in 1Y compared with 1W (Table S3). The volcanic map provided the fold change of gene expression and the significant degree of the results (Figure 2a). It was shown that the distribution of upregulated genes was greater than that of downregulated genes. The heat map facilitated understanding of the changes in gene expression (Figure 2b). It was shown that most of the genes with higher expression levels in 1Y had lower expression levels in 1W.

**Figure 3:**
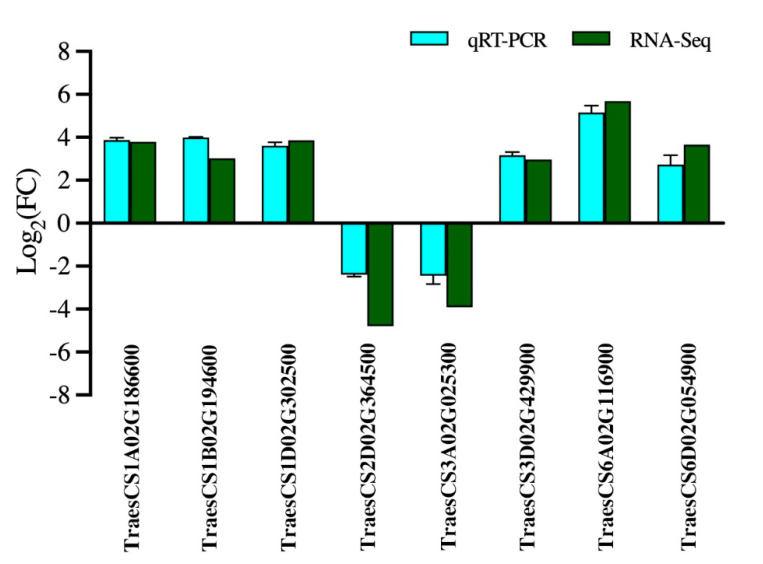
Transcript levels of selected genes and the corresponding expression data of RNA-Seq. The cyan columns represent relative expression obtained by qRT-PCR, and the green columns represent relative expression obtained by RNA-Seq.

To confirm the reliability of the RNA-Seq data, we randomly selected eight transcripts with differential expression levels between 1Y and 1W wheat NILs at the anthesis stage for qRT-PCR analysis. The results showed that the qRT-PCR analysis was in line with the RNA-Seq results (Figure 3), and the Pearson coefficient was 0.97 (Figure S2). Consequently, the qRT-PCR analysis showed that the RNA-Seq data were reliable.

### 3.4. GO and KEGG Analysis of DEGs

Gene ontology (GO) enrichment analysis was performed to understand the main biological functions of DEGs. A total of 5828 Terms (Table S4) were annotated through the GO enrichment analysis, of which 3702, 538, and 1588 terms belonging to the biological process (BP), cellular component (CC), and molecular function (MF), respectively. In the BP category, “cellular process” (GO: 0009987) and “metabolic process” (GO: 0008152) had the largest number of genes, of which 2651 and 2198, respectively; “cell part” (GO: 0044464, 4006 DEGs) and “intracellular part” (GO: 0044424, 2796 DEGs) had the largest number of genes in the CC category; the term with the largest number of genes in the MF category was “catalytic activity” (GO: 0003824, 3094 DEGs) and “ion binding” (GO: 0043167, 2284 DEGs).

**Figure 4:**
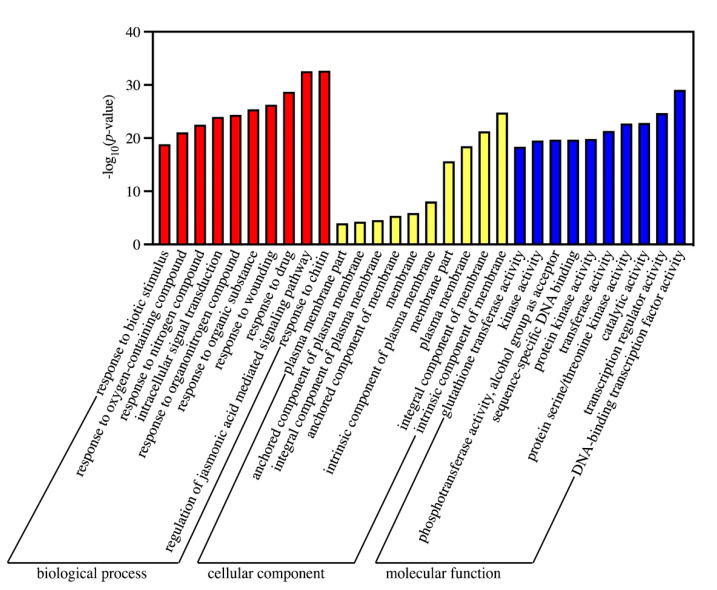
The top ten enrichment GO terms (*p* < 0.05) analysis between 1Y and 1W. The *x*-axis is the name of GO terms; the *y*-axis represents the significantly enriched *p*-values with the logarithm of base 10.

A total of 593 terms (Table S4) were significantly enriched (FDR < 0.05). The top 10 GO terms with the most significant enrichment were shown in Figure 4. In the BP category (366), “response to chitin” (GO: 0010200), “regulation of jasmonic acid mediated signaling pathway” (GO: 2000022), and “response to drug” (GO: 0042493) were the main terms. The main terms of the CC category (10) were “intrinsic component of membrane” (GO: 0031224), “integral component of membrane” (GO: 0016021), and “plasma membrane” (GO: 0005886). Among the MF category (217), “DNA-binding transcription factor activity” (GO: 0003700), “transcription regulator activity” (GO: 0140110), and “catalytic activity” (GO: 0003824) were the main terms.

**Figure 5:**
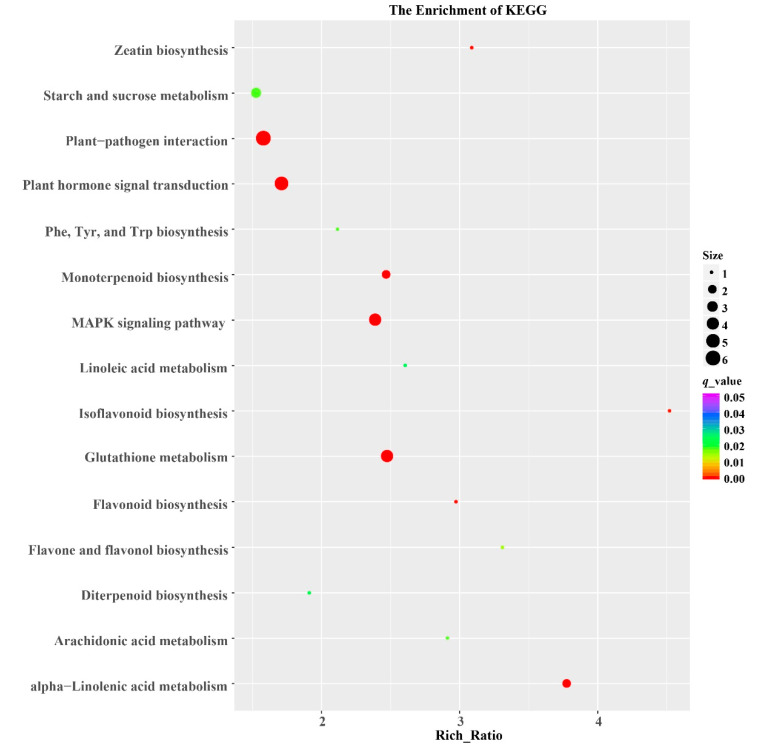
Top fifteen KEGG pathways for high and low-NUE wheat NILs grown under normal N conditions. The *x*-axis indicates the rich ratio, the *y*-axis represents the name of the KEGG pathway. The sizes of bubbles represent the number of DEGs in the corresponding pathway, and the dot color indicates the *q*-value.

We further explored the biological functions of the DEGs in different metabolic pathways by mapping them to the KEGG database. In leaves, a total of 1794 DEGs were assigned to 129 KEGG pathways in 1Y vs. 1W comparison. In the current study, DEGs were significantly enriched in “Glutathione metabolism” (map00480), “Phenylalanine, tyrosine and tryptophan biosynthesis” (map00400), and “Starch and sucrose metabolism” (map00500) according to KEGG pathway analysis (Figure 5). Glutathione S-transferases (GSTs) involved in glutathione metabolism can directly scavenge peroxides to reduce the accumulation of reactive oxygen species (ROS) [35]. In this study, 58 DEGs encoding *GSTs* were upregulated in the leaves of 1Y but not in 1W, indicating 1Y had a higher ability to scavenge excess ROS. In addition, 18 genes responsible for “Phenylalanine, tyrosine, and tryptophan biosynthesis” were differentially expressed, in which transcript levels of 15 DEGs were upregulated in 1Y. 35 upregulated DEGs related to starch and sucrose metabolism were identified in the leaves of high-NUE genotype 1Y and 16 DEGs were upregulated in low-NUE genotype 1W.

### 3.5. Expression Analysis of Genes Associated with Nitrogen Metabolism

Several known genes related to nitrogen absorption and assimilation in 1Y were identified by RNA-Seq, which was different from those in 1W. In this study, 12 DEGs encoding nitrate transporters were identified between 1Y and 1W (Table 2), of which 6 DEGs were upregulated, and six DEGs were downregulated. Nevertheless, the upregulated expression levels of genes were higher than that of downregulated genes. Besides, 4 *AMT* genes encoding ammonium transporters were upregulated in 1Y (Table 2). Moreover, two and one DEGs encoding GS and GOGAT enzymes, respectively, were upregulated and their relative expression level was higher in 1Y than in 1W except for one gene encoding the GDH enzyme. The differentially expressed genes associated with nitrogen metabolism were selected for qRT-PCR validation (Figure S3). These results indicated that the ability of nitrate absorption and assimilation was higher in 1Y than 1W under normal N conditions.

### 3.6. Expression Profiling of Genes Related to Carbon Metabolism

As shown in Table 3, the expression of 21 DEGs associated with carbon metabolism changed between 1Y and 1W. 8 DEGs related to the photosynthesis and photosynthesis-antenna proteins were relatively upregulated in 1Y, the gene encoding photosynthetic electron transport was downregulated in 1Y compared with 1W. Moreover, 12 DEGs encoding the key enzymes in the glycolysis, TCA cycle, and the PPP were identified in 1Y and 1W, including three hexokinases, three 6-phosphofructokinase, one phosphoglycerate mutase, one enolase, one pyruvate kinase, one 6-phosphogluconate dehydrogenase, one α-oxoglutarate dehydrogenase, and one malate dehydrogenase. The selection of differentially expressed genes involved in carbon metabolism was validated using qRT-PCR (Figure S4). It was noted that the expression patterns of these DEGs were higher in 1Y than in 1W. These results indicated that the carbon metabolism capacity of 1Y was stronger than that of 1W, which was conducive to the photosynthetic production of flag leaf and provided abundant carbon sources and energy for the process of nitrogen metabolism. 

### 3.7. Regulation of Differential Gene Expression by TFs

Considering the importance of TFs in regulating gene expression, we analyzed the expression levels of TF genes in detail. In this study, a total of 298 TFs belonging to 39 different TF families have been identified according to the Plant TF Database v4.0. In the 1Y vs. 1W comparison, 198 TFs were upregulated and 100 were downregulated. The selection of differentially expressed genes mapped to the TF category was validated by qRT-PCR (Figure S5). The results showed that the transcriptional regulation mechanisms were significantly different between 1Y and 1W. It has been noted that five families containing more than 20 DEGs were identified: ERF (50), WRKY (31), GRAS (26), NAC (24), and MYB (21) (Table S5). Among all the 39 TF families, a total of 15 TF families were significantly enriched in the whole genome based on the hypergeometric distribution such as ERF, WRKY, GRAS, bHLH.

### 3.8. Expression of GS, GOGAT, and GDH Gene Families in Wheat Seedlings

A total of three DEGs obtained from RNA-sequencing were selected and qRT-PCR was performed between 1Y and 1W wheat seedlings under N-deficient (N0) and normal conditions (N1). It was observed that the expression pattern of *GS* in the leaves of 1Y was 1.4 times significantly higher (*p* < 0.05) than 1W in the N1 group, whereas no significant differences in the expression levels were observed between 1Y and 1W wheat seedling leaves in the N0 group (Figure S6a). The expression of *GOGAT* was upregulated by 3.6-fold in 1Y (*p* < 0.05) in comparison with 1W in the N1 group. However, no change in expression levels was detected in the N0 group (Figure S6b). The expression of *GDH* was upregulated by 2.1-fold in the leaves of 1Y (*p* < 0.05) in the N1 group compared with 1W, whereas in the N0 group, no change in expression levels was observed (Figure S6c). The results suggest that 1Y wheat NIL is more efficient in N assimilation under normal N conditions.

## 4. Discussion

Nitrogen (N) is a key nutrient element for plant growth and development. However, N deficiency has become a major issue in agricultural production. Therefore, developing genotypes with high-NUE is one of the best ways to solve this problem. Studies have found a large genotypic variation among wheat genotypes for NUE [6], and it is important to understand the underlying molecular regulatory mechanism of high-NUE in plants [36]. Currently, a lot of research has been conducted on the molecular basis of plant response to N and the identification of N-responsive genes. Shi et al. [37] showed that *TaMPK14* is a mitogen-activated protein kinase (MAPK) family gene in wheat, which made a crucial contribution in mediating the N-starvation response through transcriptional regulation of NRT genes and modulation of related biological processes. Tan et al. [38] identified the transcriptomic expression patterns that contribute to ramie resilience to N deficiency between high-NUE and low-NUE ramie. Iqbal et al. [39] reported that the high NUE of CCRI-69 can be explained by the significant upregulation of N metabolism genes in comparison with the low NUE of XLZ-30 in cotton under low N conditions.

However, these studies focused on the plant’s response to low N stress. Unlike these studies that consider N deficiency, in our study, a normal N condition was performed. We observed large differences in response to normal nitrogen between two contrasting wheat (high-NUE 1Y and low-NUE 1W) NILs. Our results showed that high-NUE wheat had a higher N accumulation in comparison with low-NUE at maturity (Table 1), which may be due to its inherent molecular mechanisms. Therefore, we carried out a comprehensive comparative transcriptome analysis in high- and low-NUE wheat NILs to identify key regulatory genes. It is worth noting that our study is limited because there is only one tissue and one developmental stage. Through further studies on multiple tissues and developmental stages, we will gain a comprehensive understanding of the molecular mechanisms of N efficiency in wheat.

### 4.1. Nitrogen Metabolism

N is mainly absorbed by plants in the form of nitrate and ammonium, and nitrogen metabolism includes uptake, transport, and assimilation [40]. At least there are two transport systems: nitrate transporters and ammonium transporters [41,42]. Previous studies have found that low-affinity nitrate transporters (NRT1) play an important role in regulating nitrates during high external NO_3_^−^ concentration [43]. In the current study, the relatively higher expression of most genes encoding nitrate transporters and ammonium transporters in high-NUE wheat resulted in its higher nitrogen uptake capacity. The enzymes of nitrogen metabolism play an important role in nitrogen assimilation [44]. The activity of the glutamine synthetase (GS) enzyme can be considered as a potential marker to predict and select wheat genotypes with high-NUE [45]. Glutamate synthase (GOGAT) is a part of the GS/GOGAT cycle and participates in the main pathway of nitrogen assimilation [13]. Zhou et al. reported that the GS/GOGAT cycle promoted nitrogen remobilization and translocation in rice [46]. Glutamate dehydrogenase (GDH) can catalyze the exchange of NH_3_ to the amino group of glutamate, whereas GDH plays a small role in nitrogen metabolism [47]. In this study, we found three genes belonging to the *GS* and *GOGAT* gene families were upregulated, but the expression level of the *GDH* gene was downregulated in high-NUE wheat. Thus, these results indicated that nitrogen assimilation is adjusted by the GS/GOGAT cycle.

### 4.2. Carbon Metabolism

In photosynthetic systems, photosystem I (PSI) and II (PSII) are interconnected via the electron transport chain and cytochrome (Cyt) b6f complex [48]. Previous studies have shown that PSI acts as an energy converter [49] and PSII has the function of absorbing light and split water [50]. From our transcriptome data (Table 3), most of the DEGs were upregulated in the photosynthesis pathway, indicating that high-NUE wheat could enhance the expression of PSI- and PSII-related genes. In the “photosynthesis-antenna proteins” pathway, the light-harvesting complex proteins (LHCs) play an important role in capturing and transferring light energy in photosynthesis [51]. In the current study, the expression of Lhcb1 genes was upregulated in high-NUE wheat (Table 3). This result suggests that high-NUE wheat has higher expression levels of Lhc-related genes. Collectively, these upregulated genes led to the enhancement of the photosynthetic pathway in high-NUE wheat, which increased the photosynthetic rate.

The 6-phosphogluconate dehydrogenase (G6PD) is an important regulatory enzyme involved in the PPP, with the production of NADPH [52]. As we know, the production of NADPH also supplies the major reducing power for nitrate reduction in cells. In addition, it also contributes to the maintenance of the reduced state of glutathione [53]. In the current study, the transcription level of *G6PD* was upregulated (Table 3) in high-NUE wheat, and the enhanced *G6PD* expression may stimulate the PPP. Similarly, Quan et al. [54] found that the DEGs encoding this enzyme was upregulated in LN-tolerant wild barley under low nitrogen. Meanwhile, we also observed that the expression levels of genes encoding the TCA cycle were upregulated, which promoted this pathway in high-NUE wheat (Table 3). Similar findings were detected in other studies [55]. The enhanced TCA cycle provides more α-oxoglutarate (2-OG) for the GS/GOGAT cycle and enables a more rational distribution of nitrogen for the synthesis of different amino acids [56]. Collectively, high-NUE wheat had a higher potential to enhance carbon metabolism in comparison with low-NUE. These observations suggest that the enhancement of the TCA cycle and the PPP in high-NUE wheat can provide more C skeletons, and NADPH for nitrogen absorption and assimilation.

### 4.3. Transcription Factors (TFs)

TFs can regulate the expression of other genes in metabolic pathways [57], and many TFs have been detected to be involved in the regulation of nitrogen metabolism [58]. Curci et al. [23] used RNA-Seq analysis and identified more than 30 TF families in durum wheat under N deficiency. Transcriptome Analysis of rice seedlings has shown that 4 ERF genes are upregulated under low-N stress [53]. Studies have found that overexpression of *TaNAC2-5A* TF significantly promoted the genes encoding NRT and GS under low N conditions in wheat [59]. Similarly, overexpression of *OsMYB305* positively regulates *OsNRT2.1*, *OsNRT2.2*, and *OsNiR2* expression, thereby promoting N uptake and assimilation in rice [60]. The expression levels of WRKY TF genes were upregulated in wheat under low-N conditions [22], WRKY proteins may contribute to the formation of complex signaling networks and thus improve plant tolerance to low-N stress [23]. In this study, ERF TF families were the relatively large TF families, and we found that most TFs were upregulated in high-NUE wheat. Therefore, it may be assumed that these upregulated TFs can promote the expression of genes related to nitrogen metabolism in high-NUE wheat. Obviously, it will be important to determine the possible roles of these TFs for improving NUE in the future.

## 5. Conclusions

The availability of advancement in molecular technology has provided a new perspective on nitrogen metabolic pathways in plants. Nitrogen regulation is closely linked to carbon metabolism, RNA-seq can accurately measure transcription levels and identify genes that are involved in the interactions between the C and N metabolic pathways. Our results showed that the mechanism of high-NUE was as follows: (i) enhancing the GS/GOGAT cycle of N assimilation; (ii) activating photosynthetic assimilation ability; (iii) higher expression of genes related to glycolysis, the TCA cycle, and the PPP. This study provides a theoretical basis for the discovery of high-NUE genes in wheat.

## Figures and Tables

**Table 1:** Yield, total N accumulation, NUE of the two wheat NILs at the maturation stage.

NILs	Grain Yield (g plant^−1^)	Total N Accumulation (mg plant^−1^)	NUE (%)
1Y	9.43 ± 0.45 a	231.09 ± 15.57 a	49.33 ± 3.23 a
1W	7.72 ± 0.61 b	157.73 ± 10.07 b	33.41 ± 2.11 b

Note: Data are expressed as mean ± standard deviation (SD). Within a column, different lowercase letters indicate significant differences at 0.05 levels.

**Table 2:** Changes in the expression profiles of genes involved in nitrogen metabolism.

Group	Gene ID	Log_2_ (1Y/1W)	Description
NRT	TraesCS1B02G038700	1.24	Protein NRT1/PTR FAMILY 6.2
	TraesCS1D02G147400	−1.05	Protein NRT1/PTR FAMILY 5.2
	TraesCS1D02G269700	1.02	Protein NRT1/PTR FAMILY 5.10
	TraesCS3A02G081100	−1.39	Protein NRT1/PTR FAMILY 6.1
	TraesCS3A02G418700	2.93	Protein NRT1/PTR FAMILY 2.13
	TraesCS3D02G414300	4.09	Protein NRT1/PTR FAMILY 2.13
	TraesCS4A02G225400	−2.07	Protein NRT1/PTR FAMILY 4.3
	TraesCS5D02G419200	−1.18	Protein NRT1/PTR FAMILY 6.4
	TraesCS7A02G365100	1.07	Protein NRT1/PTR FAMILY 4.6
	TraesCS7B02G201900	−1.12	Protein NRT1/PTR FAMILY 6.3
	TraesCS7B02G262200	1.83	Protein NRT1/PTR FAMILY 4.6
	TraesCS7D02G297000	−1.15	Protein NRT1/PTR FAMILY 6.3
AMT	TraesCS1D02G296600	2.24	Ammonium transporter 2 member 1
	TraesCS4A02G352900	2.74	Ammonium transporter 3 member 2
	TraesCS5B02G520200	1.71	Ammonium transporter 3 member 2
	TraesCS5D02G519400	2.54	Ammonium transporter 3 member 2
GS	TraesCS6A02G298100	1.11	Glutamine synthetase
	TraesCS6B02G327500	1.08	Glutamine synthetase
GOGAT	TraesCS3D02G266400	1.87	Glutamate synthase 1 [NADH], chloroplastic
GDH	TraesCS2A02G389900	−1.45	Glutamate dehydrogenase 2, mitochondrial

Note: NRT, nitrate transporter; AMT, ammonium transporter; GS, glutamine synthetase; GOGAT, glutamate synthase; GDH, glutamate dehydrogenase.

**Table 3:** Genes encoding enzymes involved in carbon metabolism.

Group	Gene	Log_2_ (1Y/1W)	KEGG: Description
photosynthesis	TraesCS2A02G296400	2.68	psaO; photosystem I
	TraesCS2B02G312600	2.20	psaO; photosystem I
	TraesCS2D02G294300	2.22	psaO; photosystem I
	TraesCS2B02G395900	1.46	psbQ; photosystem II
	TraesCS2D02G220800	1.18	psbQ; photosystem II
	TraesCS3D02G366600	−3.79	petF; ferredoxin
photosynthesis-antenna proteins	TraesCS6A02G094200	5.90	Lhcb1; chlorophyll a/b binding protein 1
	TraesCS7A02G276400	3.43	Lhcb1; chlorophyll a/b binding protein 1
	TraesCS7D02G276300	4.21	Lhcb1; chlorophyll a/b binding protein 1
Glycolysis	TraesCS1A02G122800	1.62	hexokinase
	TraesCS3B02G525400	1.41	hexokinase
	TraesCS3D02G475600	1.87	hexokinase
	TraesCS1D02G346200	1.05	6-phosphofructokinase
	TraesCS3B02G126400	1.94	6-phosphofructokinase
	TraesCS3B02G311900	1.04	6-phosphofructokinase
	TraesCS7B02G086800	5.07	phosphoglycerate mutase
	TraesCS7D02G071200	1.44	enolase
	TraesCS4D02G018900	3.69	pyruvate kinase
PPP	TraesCS3D02G491400	1.28	6-phosphogluconate dehydrogenase
TCA	TraesCS2B02G094300	1.19	α-oxoglutarate dehydrogenase
	TraesCS1A02G122500	1.91	malate dehydrogenase

## Data Availability

The RNA-seq data was deposited in the National Center for Biotechnology Information Gene Expression Omnibus (GEO) repository with accession numbers GSE179179 (https://www.ncbi.nlm.nih.gov/geo/query/acc.cgi?acc=GSE179179 (accessed on 30 June 2021)).

## Supplementary Materials

The following are available online at https://www.mdpi.com/article/10.3390/biology10080787/s1, Figure S1: The correlation between the expression levels of genes for the three biological replicates; Figure S2: Comparison between the gene expressions obtained from RNA-Seq data and qRT-PCR; Figure S3: Validation of differentially expressed genes associated with nitrogen metabolism by qRT-PCR; Figure S4: Quantitative real-time PCR validation of differentially expressed genes involved in carbon metabolism; Figure S5: Quantitative real-time PCR validation of differentially expressed genes mapped to the transcription factor category; Figure S6. Gene expression of (a) GS (Glutamine synthetase), (b) GOGAT (Glutamate synthase), and (c) GDH (Glutamate dehydrogenase) in the leaves of 1W and 1Y NILs of wheat. The results shown are the means ± SD; Table S1: The primers used in quantitative real-time PCR; Table S2 Summary of RNA-seq data and reads mapping; Table S3: The FPKM value of 7023 DEGs in 1Y and 1W; Table S4: GO analysis of DEGs between 1Y and 1W in three main categories; Table S5: Transcription factors (TFs) differentially expressed.
