# Supplementary material for: Transcriptome Analysis of Two Near-Isogenic Lines with Different NUE under Normal Nitrogen Conditions in Wheat
Source: Biology (Basel). 2021 Aug 17;10(8):787. doi: 10.3390/biology10080787 (PMC8389668; doi:10.3390/biology10080787)
Supplement: Supplementary file 1 [file biology-10-00787-s001.zip › Supplementary Figures.pdf]

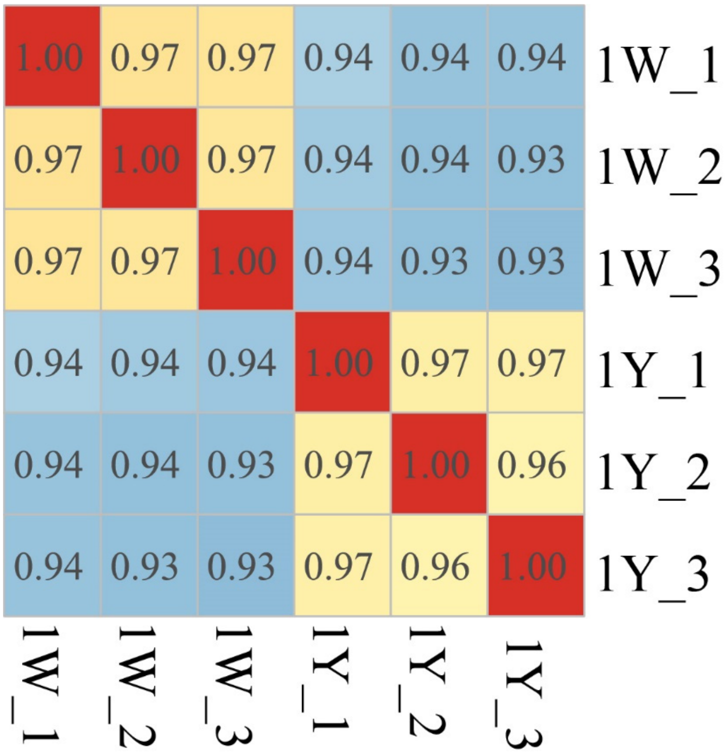

Figure S1: The correlation between the expression levels of genes for the three biological replicates.

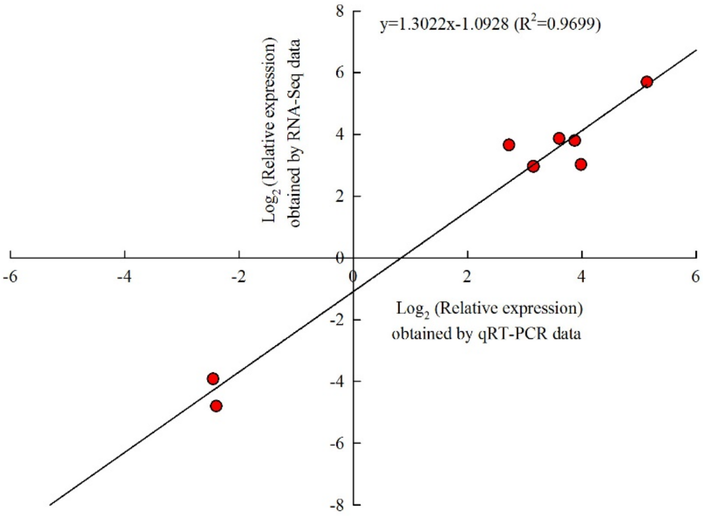

Figure S2: Comparison between the gene expressions obtained from RNA-Seq data and qRT-PCR. Each point represents a DEG, the x-axis stands for gene expression fold change in qRT-PCR, and the y-axis stands for gene expression fold change in RNA-seq.

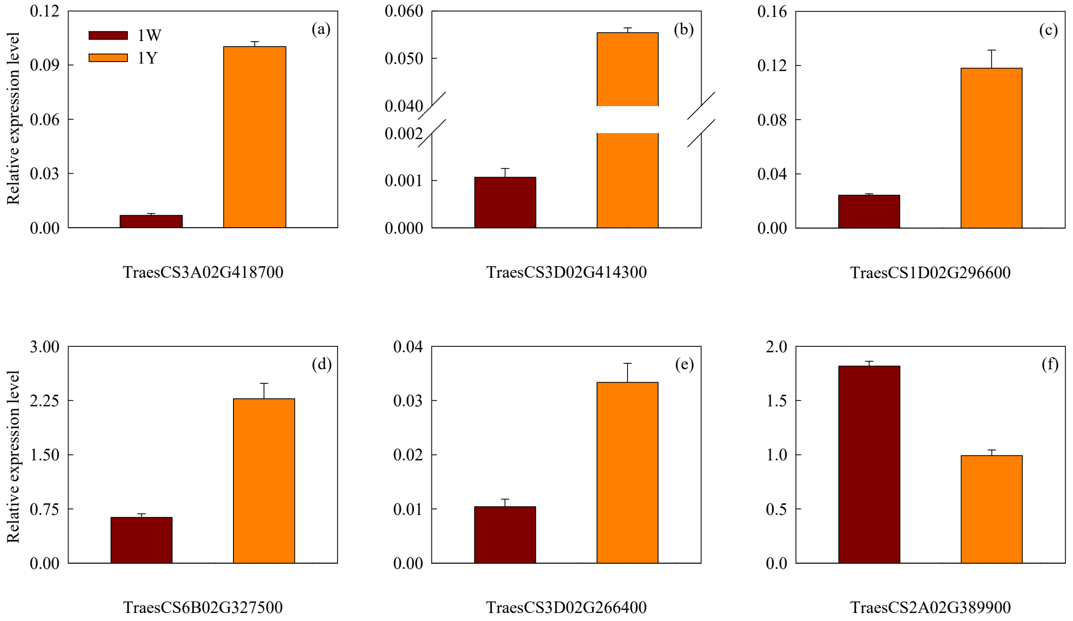

Figure S3. Validation of differentially expressed genes associated with nitrogen metabolism by qRT-PCR. Y-axis stands for relative expression level compared with GAPDH. Genes are (a) Protein NRT1/ PTR FAMILY 2.13; (b) Protein NRT1/ PTR FAMILY 2.13; (c) Ammonium transporter 2 member 1; (d) Glutamine synthetase; (e) Glutamate synthase 1; (f) Glutamate dehydrogenase 2. Data represent the means ± standard deviation (*n* = 3).

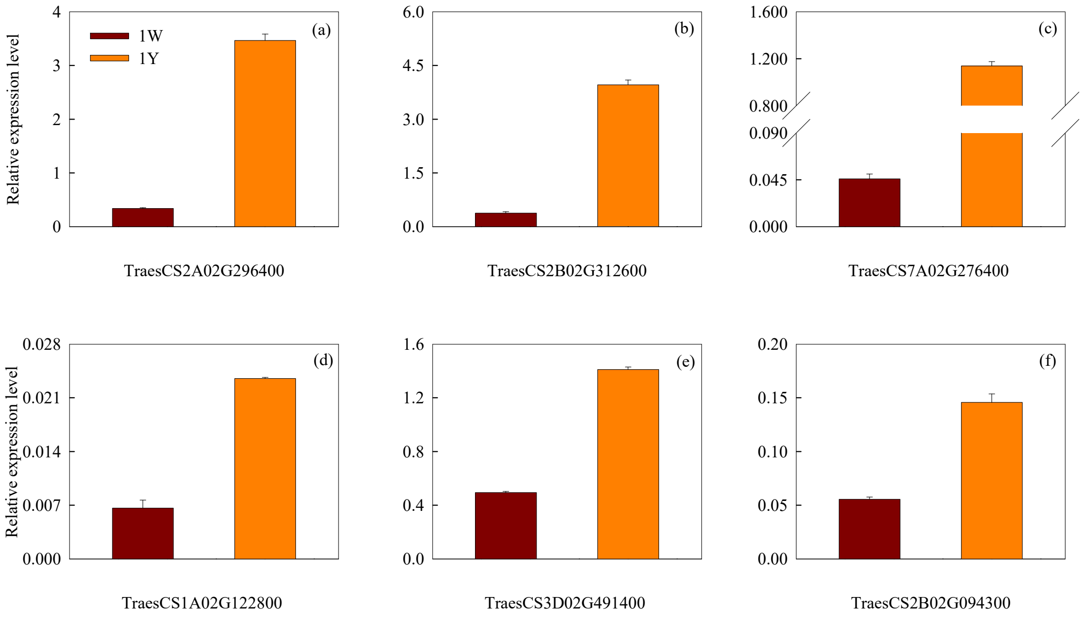

Figure S4. Quantitative real-time PCR validation of differentially expressed genes involved in carbon metabolism. Y-axis stands for relative expression level compared with GAPDH. Genes are (a) psaO; photosystem I; (b) psaO; photosystem I; (c) LHCB1; chlorophyll a/b binding protein 1; (d) hexokinase; (e) 6-phosphogluconate dehydrogenase; (f) α-oxoglutarate dehydrogenase. Data represent the means ± standard deviation (*n* = 3).

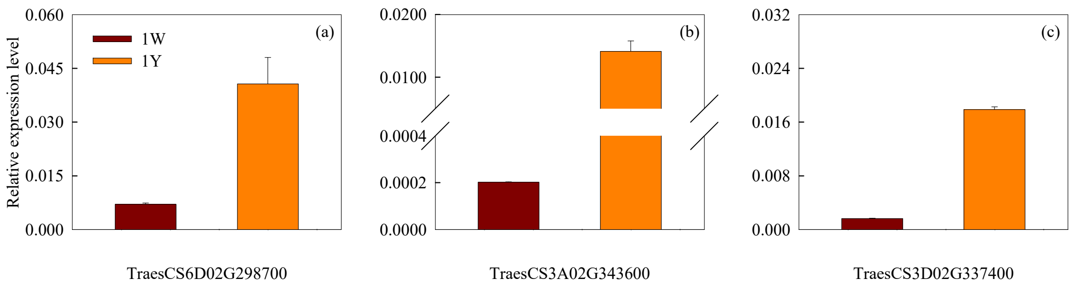

Figure S5.Quantitative real-time PCR validation of differentially expressed genes mapped to the transcription factor category. Y-axis stands for relative expression level compared with GAPDH. Genes are (a) Ethylene-responsive transcription factor ERF112; (b) WRKY transcription factor 70; (c) WRKY transcription factor 41; Data represent the means ± standard deviation (n = 3).

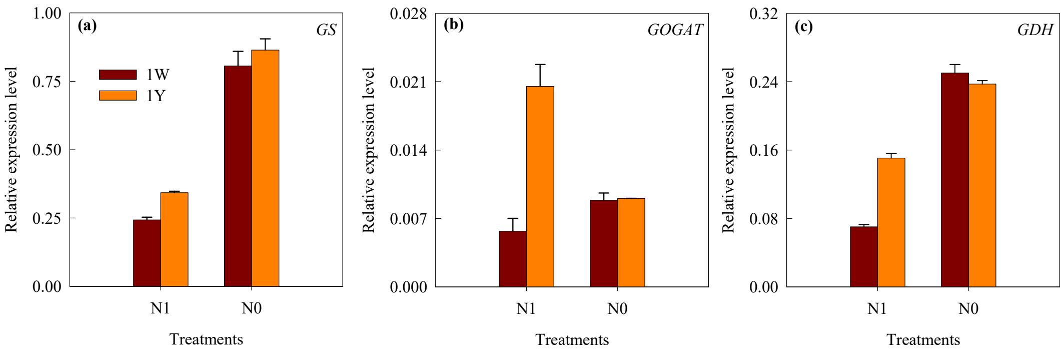

Figure S6. Gene expression of (a) GS (Glutamine synthetase), (b) GOGAT (Glutamate synthase), and (c) GDH (Glutamate dehydrogenase) in the leaves of 1W and 1Y NILs of wheat. The results shown are the means ± SD.
